# Cerebral Hemodynamics in Speech-Related Cortical Areas: Articulation Learning Involves the Inferior Frontal Gyrus, Ventral Sensory-Motor Cortex, and Parietal-Temporal Sylvian Area

**DOI:** 10.3389/fneur.2018.00939

**Published:** 2018-11-01

**Authors:** Naomi Nakamichi, Kouichi Takamoto, Hiroshi Nishimaru, Kumiko Fujiwara, Yusaku Takamura, Jumpei Matsumoto, Makoto Noguchi, Hisao Nishijo

**Affiliations:** ^1^Department of Oral and Maxillofacial Surgery, Graduate School of Medicine and Pharmaceutical Sciences, University of Toyama, Toyama, Japan; ^2^System Emotional Science, Graduate School of Medicine and Pharmaceutical Sciences, University of Toyama, Toyama, Japan

**Keywords:** articulation learning, cerebral hemodynamics, inferior frontal gyrus, ventral sensory-motor cortex, area Spt, near-infrared spectroscopy

## Abstract

Although motor training programs have been applied to childhood apraxia of speech (AOS), the neural mechanisms of articulation learning are not well understood. To this aim, we recorded cerebral hemodynamic activity in the left hemisphere of healthy subjects (*n* = 15) during articulation learning. We used near-infrared spectroscopy (NIRS) while articulated voices were recorded and analyzed using spectrograms. The study consisted of two experimental sessions (modified and control sessions) in which participants were asked to repeat the articulation of the syllables “i-chi-ni” with and without an occlusal splint. This splint was used to increase the vertical dimension of occlusion to mimic conditions of articulation disorder. There were more articulation errors in the modified session, but number of errors were decreased in the final half of the modified session; this suggests that articulation learning took place. The hemodynamic NIRS data revealed significant activation during articulation in the frontal, parietal, and temporal cortices. These areas are involved in phonological processing and articulation planning and execution, and included the following areas: (i) the ventral sensory-motor cortex (vSMC), including the Rolandic operculum, precentral gyrus, and postcentral gyrus, (ii) the dorsal sensory-motor cortex, including the precentral and postcentral gyri, (iii) the opercular part of the inferior frontal gyrus (IFGoperc), (iv) the temporal cortex, including the superior temporal gyrus, and (v) the inferior parietal lobe (IPL), including the supramarginal and angular gyri. The posterior Sylvian fissure at the parietal–temporal boundary (area Spt) was selectively activated in the modified session. Furthermore, hemodynamic activity in the IFGoperc and vSMC was increased in the final half of the modified session compared with its initial half, and negatively correlated with articulation errors during articulation learning in the modified session. The present results suggest an essential role of the frontal regions, including the IFGoperc and vSMC, in articulation learning, with sensory feedback through area Spt and the IPL. The present study provides clues to the underlying pathology and treatment of childhood apraxia of speech.

## Introduction

Producing speech is unique to humans, and is controlled by distributed interactive neural networks of cortical and subcortical structures. These speech-related networks are known to include the posterior inferior frontal gyrus (Broca's area), ventral sensory-motor cortex (vSMC), supplementary motor area, superior temporal gyrus (STG) including the auditory cortex, left posterior planum temporale (area Spt), anterior insula, basal ganglia, thalamus, and cerebellum (1–3). These neural networks, and particularly the vSMC, underlie the precise movement control of different parts of the vocal tract (i.e., the articulators, such as the tongue and lips) as well as the larynx and thoracic respiration, and do so by controlling over 100 muscles (4–6). Despite the high complexity of speech motor control, most children, but not non-human primates, can effortlessly master the skill to speak (7, 8).

Speech sound disorders include deficits in articulation (motor-based speech production) and/or phonology (knowledge and use of the speech sounds and sound patterns of language) (9), and can disturb the intelligibility of speech. The prevalence of speech sound disorders in young children has been reported to be 2–25% (9–11). In particular, apraxia of speech (AOS), which is a sub-categorical sound speech disorder, has been ascribed to brain deficits in speech motor planning/programming (with intact speech-related muscles) (12, 13), by which linguistic/phonological code is converted into spatially and temporally coordinated patterns of muscle contractions for speech production (14). To promote reorganization and plasticity in the articulation-related brain areas, various behavioral intervention programs have been applied to treat speech sound disorders (15). For children with AOS, motor training programs with the same principles as those for limb training have been applied, and have been shown to be effective (16, 17). These findings suggest that training-induced improvements of articulation might be attributed to changes in articulation-related brain areas. However, few previous imaging studies have investigated brain activity changes during actual articulation learning.

The cortical loci for articular learning remain largely unknown. However, it has been suggested that the primary motor cortex is involved in the motor learning of extremities (18–20). Furthermore, conceptual and computational models of speech production suggest that articulation is controlled online using peripheral feedback signals by way of the temporal and parietal cortices (21, 22). Based on these findings, we hypothesized that neural circuits consisting of the vSMC, temporal cortex, and parietal cortex might be involved in articular learning.

To investigate the cortical neural mechanisms of articulation learning, we recorded cerebral hemodynamic activity in healthy subjects who performed articulation learning. Recordings were made using near-infrared spectroscopy (NIRS), and we analyzed the relationships between changes in cerebral hemodynamic activity and articulation performance. NIRS is a non-invasive neuroimaging technique that can measure changes in oxygenated-hemoglobin (Oxy-Hb), deoxygenated-hemoglobin (Deoxy-Hb), and total hemoglobin (Total-Hb) in the cerebral cortex that are associated with local cortical neuronal activity (23). Although NIRS cannot detect subcortical activities, NIRS can be applied with less body and head restriction in a relatively larger space compared with the other imaging methods such as functional magnetic resonance imaging (fMRI) and positron emission tomography (PET), and less sensitive to artifacts induced by mouth movements. Thus, NIRS allows us to measure brain activity under conditions similar to actual clinical environments; we also took simultaneous acoustic measurements of produced sounds during the articulation learning.

## Methods

### Subjects

A total of 15 healthy subjects participated in the present study (mean age ± SE: 25.7 ± 1.6 years; 7 male and 8 female). Inclusion criteria were as follows: (1) right-handed according to the Japanese version of the FLANDERS handedness questionnaire (24), (2) native Japanese speaker, (3) no abnormality in dental occlusion, (4) no speech sound disorder, and (5) no experience with articulation training. All subjects were treated in strict compliance with the Declaration of Helsinki and the United States Code of Federal Regulations for the protection of human participants. The experiments were conducted with the full written consent of each subject, and the protocol was approved by the ethical committee of the University of Toyama.

### General experimental procedure

The subjects sat in a comfortable chair and were fitted with a NIRS head cap. The study consisted of two experimental sessions, which were as follows: (1) a modified condition session that involved articulation with an interocclusal splint, which was used to increase the vertical dimension of occlusion to mimic conditions of articulation disorder, and (2) a control condition session without an interocclusal splint. Each experimental session had 10 cycles; in each cycle, subjects were instructed to repeat the articulation of a Japanese shout “i-chi-ni” consisting of 3 syllables at an approximate rate of one production of the shout per second for 10 s, followed by a resting period of 60 s. The order of the sessions was randomized and counterbalanced across the subjects. Both sessions were conducted on the same day with a 10 min interval between the two sessions.

### Characteristics of the speech sound used in the study

In “i-chi-ni,” the articulatory point of the consonants of both “chi” and “ni” is the alveolar ridge, but their articulation is different; “chi” is an affricate, while “ni” is a nasal sound. The vowel included in the syllables “i-chi-ni” is “I;” in Japanese, “i” is a front vowel that is produced when the frontal surface of the tongue approaches the hard palate to the very limit where no affricate sounds occur, and is generated when the tongue is positioned at the highest position among the basic Japanese vowels. Therefore, changes in the vertical dimension of occlusion are likely to cause vocalization errors of the vowel “i” and words that include “i.”

In clinical cases where the vertical dimension of occlusion is increased by wearing a dental prosthesis or by tongue resection, it is difficult to elevate the tongue surface, and articulation of the vowel “i” and syllables containing “i” is prone to impairment. Second, utterance of “i” could minimize activity of the temporal muscles when measuring brain activity with NIRS, because, of all Japanese vowels, this vowel requires the narrowest opening of the width of the mouth, and requires less mandibular movement. Third, “i-chi-ni,” which is Japanese for “one two,” is used frequently in everyday speech. Therefore, it is easy for healthy people to articulate these syllables in a natural condition, and cognitive load other than articulatory learning is supposed to be low during conditions under which mouth movements are disturbed by an artificial attachment to the teeth.

### Interocclusal splint

To increase the vertical dimension of occlusion by 8 mm, an interocclusal splint was attached to the maxillary teeth in the present study. The body of the device was made of plastic thermoforming sheet (ERKODENT, Germany), and individually made according to the teeth size, shape, and alignment of each subject. The interocclusal splint was made in the dental clinic of the university hospital before the experiments.

### Assessment of the articulated sounds

Articulated sounds were recorded using a microphone (AT9942, Audio-Technica, Inc., Tokyo) and digitally stored on a computer hard disk at a sampling rate of 44.1 kHz with 16-bit precision. Recorded and digitized sound data were converted into spectrograms and analyzed using a commercial software (Acoustic Core 8; Arcadia, Inc., Osaka). Three independent raters, who were clinically experienced speech-language-hearing therapists, inspected the spectrograms of the articulated sounds. The raters assessed deficient voice bars on the spectrogram of [i], which was defined as vowel reduction (error). The error rates (number of errors/shout) in individual cycles in the control and modified sessions were computed.

Concordance of mean error rates (numbers of errors/shout) across 10 cycles of articulation in the control and modified sessions across the three raters was assessed using an intraclass correlation coefficient (ICC). ICC estimates and their 95% confidence intervals were calculated based on a mean-rating (number of raters = 3), absolute-agreement, and two-way random-effects model (25). Concordance was evaluated as follows: poor reliability if ICC < 0.5, moderate reliability if ICC ranged between 0.5 and 0.75, good if ICC ranged between 0.75 and 0.9, and excellent reliability if ICC > 0.9 (25). Since ICC was larger than 0.8 in the present study (see Results), the mean error rate (number of errors/shout) was computed across the three raters in each cycle in each subject, and used for later analyses.

To assess the effects of articulation learning, 10 cycles were divided into early and late phases consisting of 5 initial and 5 final cycles, respectively. The mean error rate (number of errors/shout) across 5 cycles in each phase in each experimental session was analyzed using a repeated-measures two-way analysis of variance (ANOVA).

### Hemodynamic measurement and analysis

**Figure 1:**
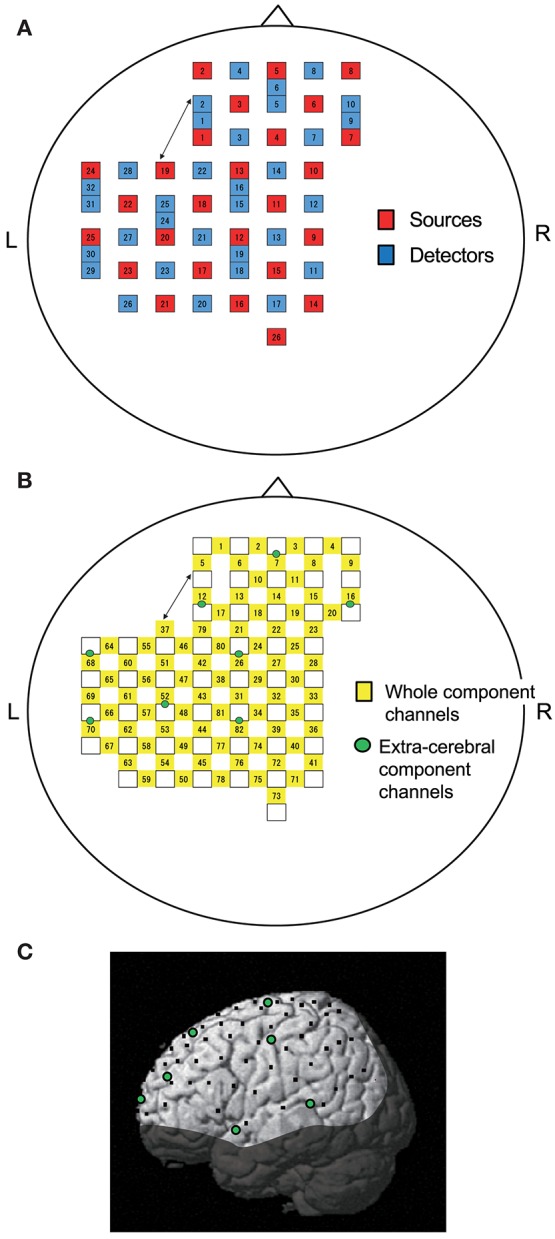
Location of NIRS probes and channels. **(A)** Arrangement of probes (sources and detectors). L, left; R, right. **(B)** Arrangement of NIRS channels. Green circles indicate extra-cerebral component channels. **(C)** 3D locations of NIRS channels. The coordinates of each NIRS channel are normalized to the Montreal Neurological Institute space using virtual registration. Black dots indicate the mean coordinates of the channels across subjects. Green circles indicate the mean coordinates of extra-cerebral component channels across subjects. The shaded area indicates the brain regions not covered by NIRS channels.

Two NIRS systems (OMM3000, Shimadzu Inc., Kyoto) were used to measure cerebral hemodynamics. Twenty-six light-source probes and thirty-two light-detector probes were placed on the head cap (Figure 1A). The bottom horizontal line of the probes was placed on the Fp1–Fp2 line according to the 10–20 EEG system. Three different wavelengths (708, 805, and 830 nm) with a pulse width of 5 ms were used to detect hemodynamic responses. The mean total irradiation power was <1 mW. Changes in Hb concentration (Oxy-Hb, Deoxy-Hb, and Total-Hb [Oxy Hb + Deoxy Hb]) were estimated based on a modified Lambert-Beer law (26, 27).

Information included in hemodynamic signals depends on probe distance between light sources and detectors (28–30). The hemodynamic signals from probes with a long distance (more than 3 cm) include not only cerebral (brain) components, but also extra-cerebral (scalp, skull, cerebrospinal fluid) components, while signals measured by probes with a short distance (<1.5 cm) mainly reflect extra-cerebral components. In the present study, multi-distance probe arrangement was applied to remove artifacts and extract cerebral hemodynamics from the whole hemodynamic responses that included both extra-cerebral and cerebral components.

The 22 detector probes were positioned 3 cm from source probes. The midpoints between source and detector probes and signals from those probes were called “whole component channels” and “whole signals,” resulting in a total of 82 channels and corresponding signals. Another 8 detector probes were positioned 1.5 cm from source probes, and the midpoints between the probes and signals from those probes were called “extra-cerebral component channels” and “extra-cerebral signals,” resulting in a total of 8 channels and corresponding signals (Figure 1B). After recording, 3D locations of the NIRS probes were measured using a Digitizer (Real NeuroTechnology Co. Ltd., Japan) with reference to the nasion and bilateral external auditory meatus.

To identify the anatomical locations of the NIRS channels in each subject, the 3D locations of the NIRS probes and channels in each subject were spatially normalized to a standard coordinate system using the software; the coordinates for each NIRS channel were normalized to Montreal Neurological Institute (MNI) space using virtual registration (31) (Figure 1C). We then identified the anatomical locations of the NIRS channels of each subject using MRIcro software (www.mricro.com, version 1.4).

### Analysis of NIRS data

To estimate the cerebral component of NIRS signals (Oxy-Hb, Deoxy-Hb, and Total-Hb), simple-subtraction methods were applied (32); the cerebral hemodynamic activity was calculated by subtraction of the extra-cerebral signals, located nearest to corresponding whole signals, from the whole signals. The subtracted NIRS signals were filtered with a bandpass filter (0.01–0.1 Hz) to remove long-term baseline drift and physiological noise induced by cardiac or respiratory activity (33, 34).

To analyze the time course of hemodynamic responses (Oxy-Hb, Deoxy-Hb, and Total-Hb) during articulation, 10 cycles were divided into early- and late-phase blocks consisting of 5 initial and 5 final cycles, respectively. The NIRS data were summed and averaged for the 5 cycles in each block. The averaged responses were corrected for baseline activity from −5 to 0 s before articulation.

To assess cortical activity, we analyzed Oxy-Hb data, since previous studies have reported that Oxy-Hb is correlated with fMRI BOLD signals (35, 36), and that Oxy-Hb may be the most consistent parameter for cortical activity (37). The cerebral Oxy-Hb data were analyzed based on a mass univariate general linear model (GLM) using NIRS SPM software (statistical parametric mapping: https://www.nitrc.org/projects/nirs_spm/, version 4.1) (38). To analyze the effects of articulation learning on cerebral hemodynamic activity, 10 cycles were divided into early- and late-phase blocks consisting of 5 initial and 5 final cycles, respectively. In the group analysis, the SPM t-statistic maps that were superimposed on the standardized brain according to the MNI coordinate system were generated based on two contrasts, as follows: task (articulation)-related cerebral hemodynamic activity in the early and late blocks, and learning-related cerebral hemodynamic activity between the two blocks. Thus, the contrast maps were as follows: articulation-related images in the early phase, articulation-related images in the late phase, and learning-related contrast images between the late and early phases in the control session, and the same maps in the modified session. Statistical significance was set at an uncorrected threshold of *P* < 0.05.

We also analyzed the correlation between hemodynamic activity during articulation and the error rate (mean number of errors/shout) in individual cycles in the modified session using simple regression analysis. First, the seven brain regions that showed significant activation in the group analysis of comparison between the early and late phases in the modified session were selected as regions of interest (ROIs). The anatomical location and MNI coordinates [(X, Y, Z) mm] of each ROI were as follows: ROI 1 {left opercular part of the inferior frontal gyrus, L-IFGoperc [MNI coordinates: (−60, 15, 23) mm]}, ROI 2 {L-IFGoperc [MNI coordinates: (−60, 8, 10) mm]}, ROI 3 {left precentral gyrus, L-PreCG [MNI coordinates: (−60, 3, 26) mm]}, ROI 4 {left Rolandic operculum, L-ROL [MNI coordinates: (−60, −3, 12) mm]}, ROI 5 {left postcentral gyrus, L-PoCG [MNI coordinates: (−60, −6, 26) mm]}, ROI 6 {L-PoCG [MNI coordinates: (−60, −10, 22) mm]}, and ROI 7 {left supramarginal gyrus, L-SMG [MNI coordinates: (−60, −30, 35) mm]}. Second, *T*-values of the group analysis in each ROI in individual cycles were calculated. Finally, in each ROI, linear regression analysis was performed between *t*-values and the error rates (numbers of errors/shout) across the 10 cycles.

### Statistical analysis

Normality of the data was evaluated using the Shapiro-Wilk test. The homogeneity of variance was evaluated using Levene's test. All statistical analyses were performed using SPSS statistical package version 19.0 (IBM Inc., New York, USA). Values of *P* < 0.05 were considered statistically significant.

## Results

### Behavioral changes

**Figure 2:**
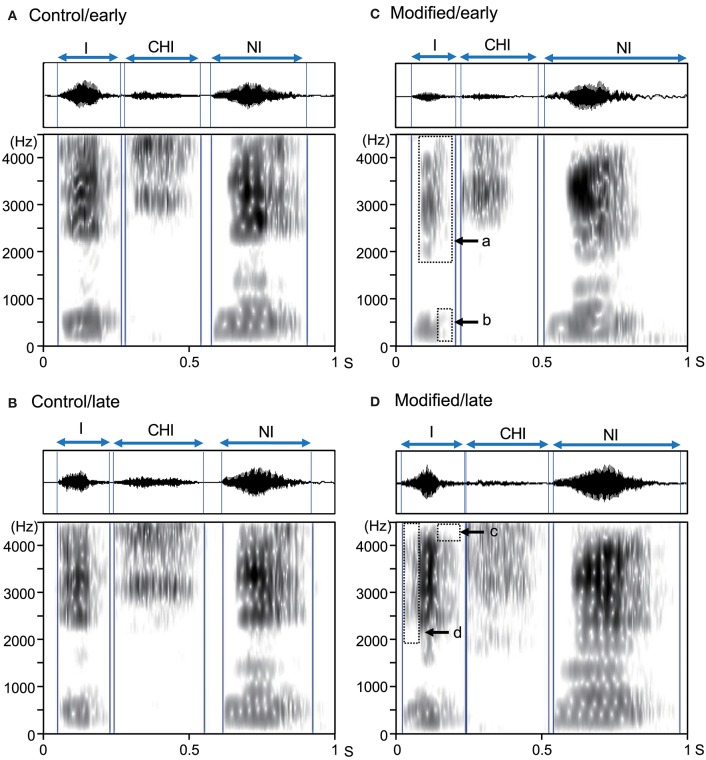
Examples of acoustic waveforms and spectrograms of articulated sounds in an example subject. **(A,B)** the early **(A)** and late **(B)** phases of the control session. **(C,D)** the early **(C)** and late **(D)** phases of the modified session. Blue vertical lines indicate the onset and offset of each syllable. Arrows (a–d) indicate vowel reduction (errors). These data were derived from the same subject.

Assessment of the all spectrograms of the 15 subjects were consistent across the 3 raters; the ICC value for mean error rate (number of errors/shout) in the control session was 0.94 (95% confidence interval [CI] = 0.86, 0.98), while the ICC value for the mean error rate in the modified session was 0.88 (95% CI = 0.72, 0.96). Figure 2 shows examples of the acoustic waveforms and spectrograms of articulated sounds in the early and late phases of the control (without an interocclusal splint) and modified (with an interocclusal splint) sessions in one subject. In the control session, no error was observed in the early (Figure 2A) and late (Figure 2B) phases. However, in the modified session, errors were observed in the early (Figure 2C) and late (Figure 2D) phases.

**Figure 3:**
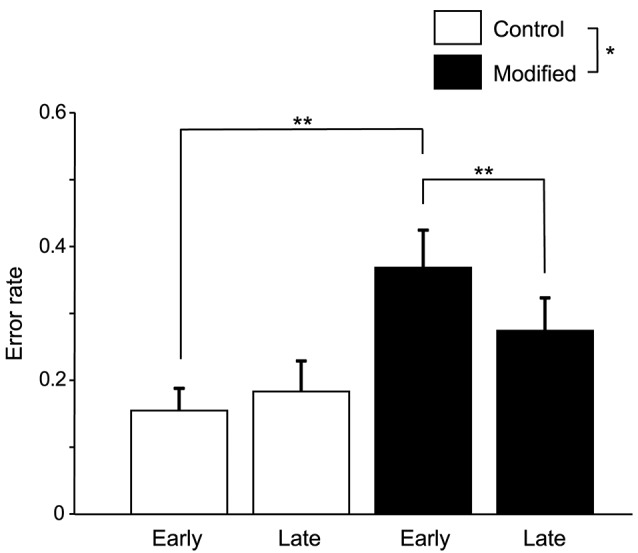
Comparison of the error rates (number of errors/shout) between the control and modified sessions. Error bars indicate the SEM. In the modified session, the error rate was significantly lower in the late phase than in the early phase. *P < 0.05, **P < 0.01.

Figure 3 shows the comparisons of the error rates (numbers of errors /shout) in the early and late phases of the control and modified sessions. A statistical analysis of the data using a repeated-measures two-way ANOVA with “session” (control vs. modified) and “phase” (early vs. late phases) as factors indicated that there were significant main effects of session [*F*_(1, 14)_ = 5.58, *P* < 0.05] and time [*F*_(1, 14)_ = 6.57, *P* < 0.05], and a significant interaction between session and phase [*F*_(1, 14)_ = 13.74, *P* < 0.01]. *Post-hoc* multiple comparisons indicated that the error rates in the early phase were larger in the modified session than in the control session (Bonferroni test, *P* < 0.01), and that the error rates were lower in the late phase than early phase in the modified session (Bonferroni test, *P* < 0.01).

### Hemodynamic responses

**Figure 4:**
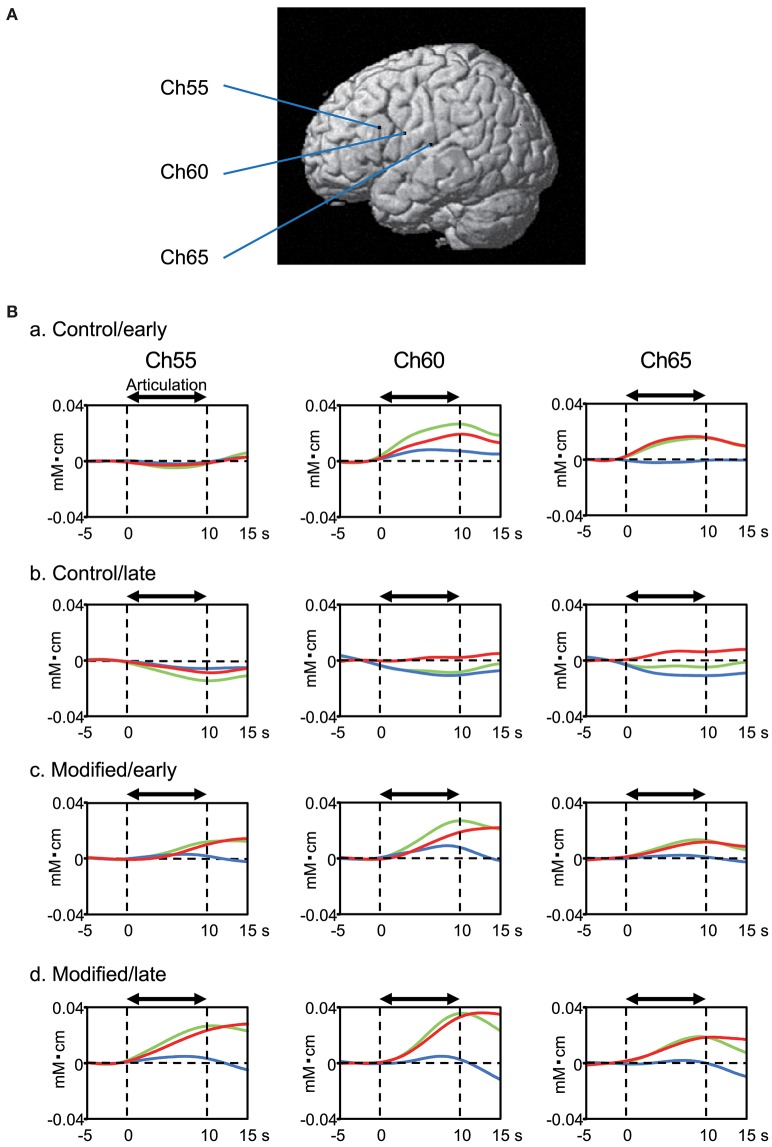
Examples of cerebral hemodynamic activity in the inferior frontal gyrus (Ch55), ventral sensory-motor cortex (Ch60), and superior temporal gyrus (Ch65) during articulation. **(A)** 3D locations of the channels. Ch55, the left opercular part of inferior frontal gyrus [MNI coordinates: (−59, 18, 23) mm]; Ch60, the left post central gyrus [MNI coordinates: (−65, 0, 20) mm]; Ch65, the left superior temporal gyrus [MNI coordinates: (−68, −17, 11) mm]. **(B)** Cerebral hemodynamic activity during articulation in the early (a) and late (b) phases of the control session, and the early (c) and late (d) phases of the modified session. Red, blue, and green lines indicate Oxy-Hb, Deoxy-Hb, and Total-Hb, respectively. The data are derived from the same subject.

Figure 4 shows examples of the cerebral hemodynamic activity during articulation in the control and modified sessions. Locations of the three cortical channels were as follows: Ch55, L-IFGoperc; Ch60, L-PoCG near the central sulcus; and Ch65, L-STG near the Sylvian fissure (Figure 4A). Oxy-Hb and Total-Hb concentrations in the L-IFGoperc, L-PoCG, and L-STG were increased during articulation (Figure 4B). There were trends of hemodynamic responses during articulation across the different experimental conditions, as follows. Hemodynamic activity during articulation was larger in the modified session than the control session. Furthermore, hemodynamic activity during articulation was larger in the late than early phases in the modified session, while it was larger in the early phase than in the late phase in the control session.

**Figure 5:**
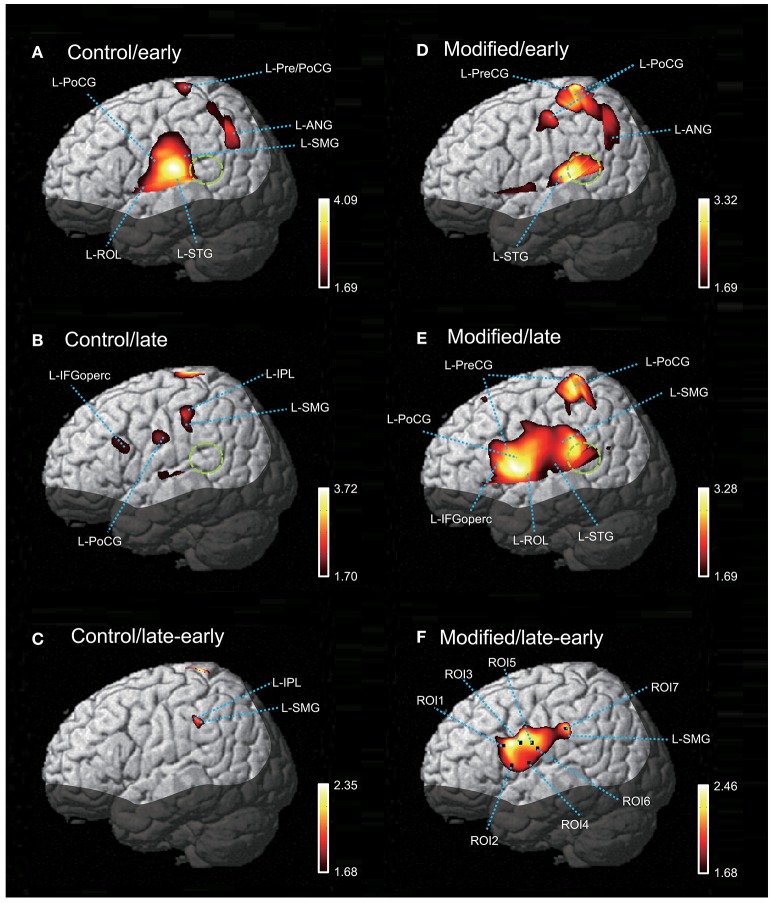
The NIRS-SPM T-statistic maps in the control **(A–C)** and modified **(D–F)** sessions. Activation map in the early **(A)** and late **(B)** phases of the control session, and in the early **(D)** and late **(E)** phases of the modified session are shown. Normalized group results of the subtraction of the early phase from the late phase in the control **(C)** and modified **(F)** sessions are also shown. Colored bars indicate the *T*-values of active voxels. Green dotted circles indicate area Spt (posterior part of the planum temporale). L-ROL, left Rolandic operculum; L-STG, left superior temporal gyrus; L-PoCG, left postcentral gyrus; L-SMG, left supramarginal gyrus; L-IFGoperc, left opercular part of the inferior frontal gyrus; L-PreCG, left precentral gyrus; L-ANG, left angular gyrus.

Figure 5 shows contrast images resulting from the group analysis based on the GLM with NIRS-SPM. In the early phase of the control session (Figure 5A), articulation-related activity was observed in the vSMC, including the L-ROL and L-PoCG, as well as in the dorsal SMC, including the L-Pre/PoCG. Furthermore, activation was observed in the temporal cortex, including the L-STG, and in the inferior parietal lobe (IPL), including the L-SMG and left angular gyrus (L-ANG). In the late phase of the control session (Figure 5B), articulation-related activity was less evident; there was activation in the L-IFGoperc, PoCG, L-SMG, and left IPL (L-IPL). The green dotted circles in Figure 5 indicate the approximate location of the posterior part of the planum temporale (posterior Sylvian fissure at the parietal–temporal boundary, area Spt) based on the data derived from (39). Almost no activation was observed in area Spt, although activation was observed in the L-STG in the control session. The contrast image map between the early and late phases in the control session revealed that only the L-SMG was significantly activated (Figure 5C).

In the early phase of the modified session (Figure 5D), activation in the vSMC was less evident; only activation in the dorsal and ventral parts of the SMC was observed. In the dorsal SMC, as well as the L-ANG, a similar articulation-related activation pattern to that in the early phase of the control session was observed in the L-Pre/PoCG and L-ANG. In the temporal cortex, significant activation was observed in the L-STG as well as area Spt (Figure 5D). In the late phase of the modified session (Figure 5E), wider activation in and around the vSMC was observed in the L-IFGoperc, L-ROL, L-PreCG, L-PoCG, and L-SMG. In the temporal cortex, activation was observed in the L-STG and area Spt (Figure 5E). The contrast image map between the early and late phases in the modified session (Figure 5F) revealed activation in the L-IFGoperc and vSMC (L-PreCG, L-PoCG, and L-ROL), as well as the L-SMG.

### Relationships between hemodynamic responses and articulation errors

**Figure 6:**
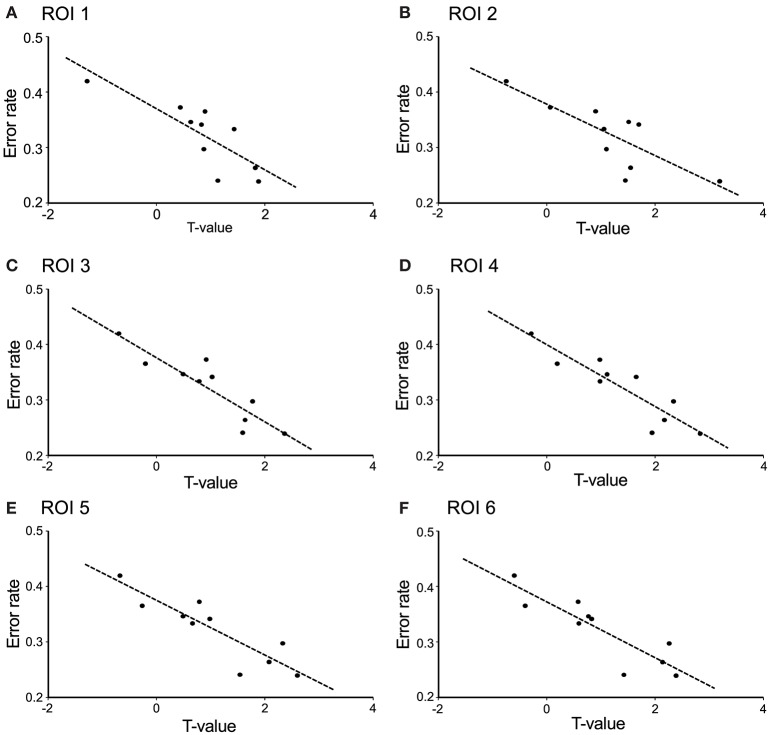
Correlation between cerebral hemodynamic activity (*T*-values) in the 6 ROIs and syllable production errors (error rates) in the modified session. The hemodynamic activity in all 6 ROIs was significantly negatively correlated with the error rates (*P* < 0.01). **(A)** ROI 1 was the left opercular part of the inferior frontal gyrus; **(B)** ROI 2 was the left opercular part of the inferior frontal gyrus; **(C)** ROI 3 was the left precentral gyrus; **(D)** ROI 4 was the left Rolandic operculum; **(E)** ROI 5 was the left postcentral gyrus; **(F)** ROI 6 was the left postcentral gyrus. The 3D locations of the 6 ROIs are shown in Figure 5F.

The contrast image map between the early and late phases in the modified session (Figure 5F) indicated significantly greater activation in L-IFGoperc, vSMC, and L-SMG in the late phase than in the early phase (see above). These results suggested that these brain areas might be involved in articulation learning. We investigated this possibility by analyzing the relationship between hemodynamic responses in these areas and the error rates (numbers of errors/shout) across the 10 cycles. Figure 6 shows the correlation between the articulation-related cortical activation (T-values) in the 6 ROIs in the L-IFGoperc and vSMC and the error rates in the modified session. The 3D locations of the ROIs are shown in Figure 5F. There were significant negative correlations between the error rates and hemodynamic responses (*T*-value) across 10 cycles in ROI 1 (L-IFGoperc) [*F*_(1, 9)_ = 15.20, *P* < 0.01; *r*^2^ = 0.66], ROI 2 (L-IFGoperc) [*F*_(1, 9)_ = 13.97, *P* < 0.01; *r*^2^ = 0.64], ROI 3 (L-PreCG) [*F*_(1.9)_ = 33.21, *P* < 0.01; *r*^2^ = 0.81], ROI 4 (L-ROL) [*F*_(1, 9)_ = 33.61, *P* < 0.01; *r*^2^ = 0.81], ROI 5 (L-PoCG) [*F*_(1, 9)_ = 28.11, *P* < 0.01; *r*^2^ = 0.78], and ROI 6 (L-PoCG) [*F*_(1, 9)_ = 26.67, *P* < 0.01; *r*^2^ = 0.77] (Figure 6). However, in the L-SMG, there were no significant correlations between error rates and hemodynamic responses (*T*-value), even in the ROI with the highest *T*-value (ROI 7) within the L-SMG [*F*_(1, 9)_ = 2.38, *P* = 0.16; *r*^2^ = 0.23).

## Discussion

### Cortical activation during articulation

In the early phase of the control session and the late phase of the modified session, articulation-related activity was observed in (i) the vSMC, including the L-ROL, L-PreCG, and PoCG, (ii) the dorsal SMC, including the L-Pre/PoCG, (iii) the L-IFGoperc, (iv) the temporal cortex, including the L-STG, and (v) the L-IPL, including the L-SMG and L-ANG. These activated areas are considered to be involved in phonological and articulatory processing.

In the vSMC, including the precentral gyrus (premotor and motor areas), postcentral gyrus (somatosensory area) and subcentral area (Rolandic operculum; both somatosensory and motor-related areas), speech-articulators (i.e., the tongue, lips, jaw, and larynx) are somatotopically represented, and these representations overlap somewhat (4–6, 40). Consistent with this previous work, we observed activation in these areas in the early phase of the control session. However, in the late phase of the control session, activation in these areas was less evident, which might be indicative of efficient processing due to adaption in the early phase. The dorsal SMC activation observed in the present study might be involved in respiratory control during syllable production, as has been noted in previous works (1, 41). In the present study, activation of the left posterior part of classic Broca's area (pars opercularis of the left inferior frontal gyrus, L-IFGoperc) was observed during articulation. It has been reported that Broca's area consists of heterogeneously functional areas; its posterior part (L-IFGoperc) is involved in phonological processing during overt speech, while its anterior part (pars triangularis and orbital part of the inferior frontal gyrus) is involved in semantic processing (42–44). The syllables used in the present study were a kind of a shout, and had little semantic meaning, which might induce selective activation in the L-IFGoperc involved in phonological processing.

The STG, including the auditory cortex, was activated in the present study. This region might respond to one's own voice, and has been reported to be important for the processing of produced phonemes or syllables to send auditory feedback to the motor system for online control of articulation (1, 2). Indeed, subjects tend to compensate their vocal production when auditory feedback is artificially altered by changing formant frequencies, introducing a delay, or masking (45–48). The L-SMG, activated in the present study, has been reported to coactivate with the IFGoperc and vSMC in various phonological processing tasks (49, 50). Furthermore, the SMG has been implicated in proprioceptive processing (51), which suggests that the SMG might also encode movements of the articulatory muscles. These findings suggest that the SMG-frontal motor (i.e., IFGoperc and vSMC) system functions as an “articulatory loop” in the dorsal pathway from the temporal auditory and somatosensory cortices to the frontal cortex through the inferior parietal lobe (SMG), in which phonemic as well as oral somatosensory information is mapped onto motor representations for articulation (52, 53). The L-ANG is also linked to the IFGoperc through the dorsal pathway and has been implicated in phonological processing, such as phoneme discrimination (54).

Area Spt showed a characteristic activation pattern; area Spt was activated in the modified session in which articulation learning was required, while most parts of area Spt were silent in the control session in which no articulation learning was required. Area Spt has been reported to be activated both during auditory perception and covert production of speech (39). Furthermore, brain damage regions near area Spt have been associated with conduction aphasia (55, 56), whereby phonemic errors are more frequent than meaning-based errors (57–59). These findings suggest that area Spt is involved in sensory-motor integration, which is essential for articulation learning (2, 21, 39).

### Neural mechanisms of overt articulation learning

In the present study, the subjects decreased speech production errors during articulation learning in the modified session without any feedback from the experimenters. This suggests that they used their own distorted sounds of the syllables and/or oral somatosensory sensation as feedback signals to improve their articulation. Based on conceptual and computational models of speech production (21, 22), articulation might have been improved using feedback signals in the following potential stages: (i) the sounds (and/or somatosensory sensation) produced by articulation are encoded by their own sensory systems and representations created from auditory (as well as somatosensory) feedback are stored in the short-term memory buffer (area Spt or inferior parietal lobe, including the SMG and ANG), (ii) the feedforward representation of articulatory motor commands are created and compared against these stored representations, and (iii) the mismatch between the two representations is used to improve future articulatory commands. Although there are two (dorsal and ventral) pathways between the temporal auditory and frontal articulatory (IFGoperc and vSMC) systems, the dorsal pathway is essential for articulatory learning (21).

In the dorsal pathway, the SMG and ANG receive inputs from the auditory cortex and have reciprocal connections with the IFGoperc and ventral premotor area in the vSMC (60–62), which is involved in articulatory motor planning (63). Since the SMG functions as a storage house of phonological short-term memory (64), feedback from the auditory system might be transferred to the frontal articulatory system through the SMG to improve articulation. Consistent with this idea, inhibition of the SMG by repetitive transcranial magnetic stimulation (rTMS) has been found to disturb adaptive responses to compensate one's own speech when auditory feedback was altered (65). Area Spt is another candidate area that connects the temporal auditory and frontal articulatory systems (2, 39). Since area Spt was activated selectively in the modified session, during which subjects had to learn new articulation, area Spt might have a more specific role in articulation learning using auditory feedback than the SMG and ANG.

In the present study, hemodynamic activity in ROIs 1–6 (IFGoperc and vSMC) was negatively correlated with articulation error rates. These findings indicate these areas to have a strong association with articulation learning. It has been suggested that the motor learning process by repeating a motor task involves the primary motor cortex, where motor learning is partly mediated through synaptic plasticity, including long-term potentiation (LTP)-like and long-term depression (LTD)-like mechanisms (18–20). The present results revealed an increase in hemodynamic activity in the late phase of the modified session; this suggests that synaptic changes such as LTP might underlie articulation learning in the IFGoperc and vSMC. It has been reported that excitability in speech-related premotor and motor areas was increased when distorted speech sounds or those with noises were presented (66–68). This suggests that the distorted sounds (i.e., sounds with errors) might have triggered synaptic plasticity in the modified session. As far as we know, the present results provide the first evidence of dynamic relative changes in the IFGoperc and vSMC during articulation learning.

On the other hand, in patients with stroke, regions associated with AOS with deficits in speech motor planning/programming (see Introduction) include the left premotor and motor areas (69), suggesting that the premotor and motor areas in the left vSMC are essential for skilled articulatory control. These results are consistent with the present results in which hemodynamic activity in the IFGoperc and vSMC was associated with articulation improvement. A computer simulation study has suggested that childhood AOS is ascribed to developmental deficits in the formation of feedforward programs to control articulators (70). These findings suggest that childhood AOS might be a result of functional deficits in the IFGoperc and vSMC. Thus, the present results provide a neuroscientific basis of articulation learning, which might be disturbed in AOS.

There are some limitations to the present study. First, the spatial resolution of NIRS is less accurate than fMRI. Second, we did not analyze activity in the cerebellum, basal ganglia, or right cerebral cortex, which are involved in speech production and motor learning (see Introduction). Third, Japanese language has some different phonological patterns from other languages as well as some similarities [e.g., (71, 72)]. Therefore, the present results obtained from native Japanese speakers may not be directly applicable to phonological learning in other languages. Further studies are required to (1) generalize our present findings across different language speakers, (2) to clarify the role of brain regions not analyzed in the present study in articulation learning, and (3) to clarify the neurophysiological mechanisms of articulation learning in the IFGoperc and vSMC. Nevertheless, the present results provide clues to the underlying pathology and treatment of AOS.

## Author contributions

NN, KT, MN, and HisN designed research. NN, KT, and KF performed research. NN, KT, HirN, JM, YT, and HisN analyzed data. NN, KT, MN, and HisN wrote the manuscript.

### Conflict of interest statement

The authors declare that the research was conducted in the absence of any commercial or financial relationships that could be construed as a potential conflict of interest.
